# *Cichorium pumilum* Jacq Extract Inhibits LPS-Induced Inflammation via MAPK Signaling Pathway and Protects Rats From Hepatic Fibrosis Caused by Abnormalities in the Gut-Liver Axis

**DOI:** 10.3389/fphar.2021.683613

**Published:** 2021-04-29

**Authors:** Chang Han, Xi Wu, Nan Zou, Yunsheng Zhang, Jinqi Yuan, Yuefeng Gao, Wen Chen, Jia Yao, Cong Li, Jinqiu Hou, Dongmei Qin

**Affiliations:** ^1^ Key Laboratory of Xinjiang Phytomedicine Resource and Utilization, Ministry of Education, School of Pharmacy, Shihezi University, Shihezi, China; ^2^ Dongfang Hospital, Beijing University of Chinese Medicine, Beijing, China; ^3^ First Affiliated Hospital, School of Medicine, Shihezi University, Shihezi, China; ^4^ Husbandry Research Institute, Xinjiang Academy of Animal Science, Urumqi, China; ^5^ State Key Laboratory of Animal Nutrition, College of Animal Science and Technology, China Agricultural University, Beijing, China

**Keywords:** liver fibrosis, *Cichorium pumilum* Jacq, gut microbiota, inflammation, lactucin

## Abstract

The development of liver fibrosis is closely related to the gut microbiota, and the “gut-liver axis” is the most important connection between the two. ethyl acetate extract of *Cichorium pumilum* Jacq (CGEA) is an herbal extract consisting mainly of sesquiterpenoids. The anti-inflammatory and hepatoprotective effects of CGEA have been reported, but the anti-fibrotic effects of CGEA via intestinal microbes and the “gut-liver axis” cycle have rarely been reported. In this study, we observed that CGEA not only directly attenuated inflammatory factor levels in inflamed mice, but also attenuated liver inflammation as well as liver fibrosis degeneration in rats with liver fibrosis caused by colitis. We observed *in vitro* that CGEA significantly promoted the growth of Bifidobacterium adolescentis. Similarly, fecal 16S rDNA sequencing of liver fibrosis rats showed that CGEA intervention significantly altered the composition of the intestinal microbiota of liver fibrosis rats. CGEA increased the abundance of intestinal microbiota, specifically, CGEA increased the ratio of Firmicutes to Bacteroidetes, CGEA could significantly increase the levels of Ruminococcus. In addition, CGEA intervention significantly protected intestinal mucosal tissues and improved intestinal barrier function in rats. Lactucin is the main sesquiterpenoid in CGEA, and HPLC results showed its content in CGEA was up to 6%. Lactucin has been reported to have significant anti-inflammatory activity, and in this study, we found that Lactucin decreased p38 kinases (p38), phosphorylation of the extracellular signal-regulated kinase (ERK) and protein kinase B (AKT) protein phosphorylation in lipopolysaccharide (LPS)-activated RAW264.7 cells, thereby reducing mRNA expression and protein expression of pro-inflammatory factors inducible nitric oxide synthase (iNOS) and cyclooxygenase-2 (COX-2), and inhibiting the release of inflammatory factors interleukin (IL)-6 and nitric oxide (NO), exerting anti-inflammatory effects. In summary, the prevention of liver fibrosis caused by intestinal inflammation by CGEA may be achieved by regulating the intestinal microbiota and restoring the intestinal barrier thereby improving the “gut-liver axis” circulation, reducing liver inflammation, and ultimately alleviating liver fibrosis. Notably, the direct anti-inflammatory effect of CGEA may be due to its content of Lactucin, which can exert anti-inflammatory effects by inhibiting the phosphorylation of Mitogen-activated protein kinase (MAPK) and Akt signaling pathways.

## Introduction

Hepatic fibrosis is a chronic liver disease that can lead to end-stage liver disease or cirrhosis and eventually to liver cancer. This causes a serious threat to human health. Extensive researches have been devoted to analyze the mechanisms underlying the pathogenesis of liver fibrosis in recent years, but there are fewer studies on the resistance to liver fibrosis from the perspective of intestinal microbiota and the “gut-liver axis” circulation.

In recent years, the relationship between gut microbes and the liver is receiving increasing attention (Bajaj et al., 2012; Ling et al., 2015). Marshall’s (Marshall, 1998) concept of the “gut-liver axis” points out that when the intestinal mucosa is damaged, the permeability of the intestine increases and harmful bacteria and their metabolites [lipopolysaccharide (LPS) and endotoxins] enter the portal vein and move to the liver with blood circulation (“leaky gut”) (Marshall, 1998; Kamada et al., 2013; Nakamoto et al., 2017), activate the liver’s immune response, and release inflammatory cytokines such as IL-6, IL-1β, TNF-α, etc., thereby accelerate the progression of liver fibrosis (Lau et al., 2015).

In addition, LPS is a key factor in triggering intestinal inflammation (Hu et al., 2016a), which can further affect liver function through the “gut-liver axis” (Li et al., 2018). Also, LPS can directly activate intrahepatic and extrahepatic mononuclear-macrophages, releasing large amounts of inflammatory factors such as nitric oxide (NO), tumor necrosis factor (TNF)-α, interleukin (IL)-1β, interleukin (IL)-6 (Ribeiro et al., 2015; Liu et al., 2017), etc., occurrence of these inflammatory events is regulated by non-receptor-type tyrosine kinases, such as Janus kinase (JAK) 2, phosphoinositide 3-kinase (PI3K), protein kinase B (Akt), and mitogen-activated protein kinases (MAPKs) (Van Dort et al., 2015; Hu et al., 2016b). Especially up-regulating activated MAPK protein expression, including extracellular signal-regulated kinases (ERK), c-Jun N-terminal kinase (JNK), and p38 are closely related to intestinal inflammation (Xie et al., 2019). As reported earlier (Zanello et al., 2011), the inflammatory response of swine intestine caused by E.*coli* infection is closely related to the MAPK signaling pathway, and the Astilbin improves inflammatory bowel disease through PI3K, STAT3, and MAPK signaling pathways (Han et al., 2020). Published work suggests that regulation of gut microbial composition, reduces the phosphorylation of MAPK and AKT proteins (Serreli et al., 2020; Zhang et al., 2020). In summary, by modulating the gut microbial composition and reducing intestinal inflammation, pathogenic bacteria and their metabolites in the liver can be inhibited, thereby reducing liver fibrosis.

*Cichorium pumilum* Jacq (CG) is a traditional herb in Uighur medicine and is often used in folklore as a remedy for liver disease (Tong et al., 2017). Substantial studies on CG were carried out by our group and it was found that *Cichorium pumilum* Jacq ethyl acetate extract (CGEA) reduces CCl_4_ and thioacetamide-induced liver fibrosis in rats via the TGF β/Smad signaling pathway (Qin et al., 2013; Qin et al., 2014). CGEA was also found to have significant growth inhibitory effects on both *Staphylococcus aureus* and *Enterococcus faecalis* (Han et al., 2019). According to Lee CG can significantly promote the growth of probiotic bacteria and reduce the growth of harmful bacteria in the intestine, indicating that CG has a significant “probiotic-like effect” (Lee et al., 2015). Therefore, we predict that CGEA may reduce inflammation by modulating intestinal microbes to combat liver fibrosis.

Studies have shown that the main components of CGEA are sesquiterpene lactones, and the main components are Lactucin and Lactucopicrin (Pyrek, 1985). Our group isolated a total of 25 compounds from CGEA, 14 of which were sesquiterpene lactones (Supplementary Table S3), and preliminary experimental results showed that Lactucin has extremely strong anti-inflammatory activity (Dang et al., 2019). Lactucin is the most abundant terpenoid in CGEA with significant antibacterial, adipogenesis-inhibiting effects (Wang et al., 2020). Although Lactucin exhibits excellent anti-inflammatory activity, however, its anti-inflammatory mechanism of action has not been much reported, so we investigated the anti-inflammatory effects of Lactucin in MAPK and AKT signaling pathways at the cellular and molecular biological levels to elucidate the mechanism of Lactucin’s anti-inflammatory effects.

On these indications, we predicted that CGEA could improve liver fibrosis through the “gut-liver axis” by reducing intestinal inflammation and promoting probiotic growth, with Lactucin playing a major anti-inflammatory role. Altogether, in the present study, we demonstrated that CGEA can promote probiotic growth and prevent liver fibrosis caused by 2,4,6-trinitrobenzen sulfonic acid (TNBS)-induced intestinal inflammation. We then validated the anti-inflammatory effect of Lactucin *in vitro* using RAW264.7 cells.

## Materials and Methods

### Preparation of CGEA and Lactucin

CGEA and Lactucin were prepared by our research team as follows: 1 kg of dried roots of CG (Xinjiang Medicines Co., Ltd., Xinjiang, China. identified by Prof. Mehmet Nur Ayhoi at Xinjiang Uygur Medical College) was soaked in 95% ethanol for 5 times at room temperature (2 L each time for 3 days), the extract was suspended in water, extracted 1:1 with ethyl acetate and water, and the solvent was volatilized at room temperature to obtain CGEA (7.644 g). The CGEA fraction was fractionated by a silica gel column chromatograph (CC) eluting with a gradient of CH_2_Cl_2_-MeOH (from 30:1 to 3:1) to give subfractions B. Fraction B was subjected to a MCI CC to remove pigment eluted excessively with 70, 80, 90 and 100% methyl alcohol to give subfractions B1. Fraction B1 was separated by an RP C18 silica gel CC to give compound 10 (38 mg), and the compound 10 was identified by^1^H NMR and ^13^C NMR spectroscopy as Lactucin (Dang et al., 2019) (Supplementary Figure S1).

### High Performance Liquid Chromatography Analysis

The composition of CGEA was analyzed using Agilent 1,290 Infinity II HPLC (Agilent, Germany), and the retention time of the detected peaks was compared to that of the Lactucin standard (The standard was prepared by the research group with a purity of ≥95%) for comparison and determination of the Lactucin composition and content in CGEA. HPLC was equipped with a (4.6 × 150 mm, 5 μm) Agilent TC-C18 liquid chromatography column (Agilent, Germany). Mobile phase: Methanol (A)-0.1% formic acid (B), Elution gradient: 0–10 min, 25% A-30% A; 10–20 min, 30% A-70% A; 20–30 min, 70% A-70% A, flow rate 1.0 ml/min, column temperature 30°C, detection wavelength 275 nm, injection volume 5 μL.

### Protocol of Animal Experiments *in vivo*

Thirty adult male Sprague Dawley (SD) rats, weighing 180 ± 10 g, were provided by the Xinjiang medical university Experimental Animal center (Xinjiang China) [No. SCXK (Xin) 2016-0004]. The rats reared in alternate light and dark environments at approximately 22°C. All experimental procedures were approved by the Ethics Committee of First Affiliated Hospital, Shihezi University School of Medicine (Approval No. 2020-035-01).

After 7 days of adaptive feeding, the rats were randomly divided into five baskets. The rats fasting the night before modeling, drinking water normally, the rats were given intraperitoneal injection of 10% chloral hydrate (ShangHai Macklin Biochemical Co., Ltd., ShangHai, China) the next day, and the rats except blank control group were inverted after anesthesia to insert a 2 mm-diameter silicone hose with solution (The blank control group was fed with normal saline and the remaining 5% 2,4,6-trinitrobenzensulphonic acid (TNBS, BeiJing OuHe Technology Co., Ltd., BeiJing, China) were mixed with 50% ethanol, 60 mg kg^−1^ TNBS) into the intestinal depth of about 8 cm from the anus. Twice a week for 12 weeks, starting at week 13, except for the blank control group, the rats were randomly divided into model group, positive group (Sulfasalazine, Shanghai Xinyi Tianping Pharmaceutical Co., Ltd., ShangHai, China), high dose group of CGEA-Ⅰ (CGEA-Ⅰ, 150 mg kg^−1^), low dose group of CGEA-Ⅱ (CGEA-Ⅱ, 100 mg kg^−1^), the dosage is set based on the previous research basis of our research team (Qin et al., 2019a). Dose groups were fed with the respective medicine. The blank control group and the model group were given normal saline of equal volume once a day for 14 days.

At the end of the experimental period, after fasting for 12 h, all rats were weighed, collected blood by abdominal aorta after anesthetization by injection with 10% chloral hydrate solution and sacrificed. Subsequently, serum was separately prepared by centrifuge at the temperature of 4°C for 15 min for biochemical detection. Rat livers and colon were collected and then a small portion of liver and intestinal tissue samples immediately dissected and fixed in 4% paraformaldehyde (Biosharp Life Sciences Technology Co., Ltd., BeiJing, China). The remaining tissues and the freshly collected feces were rapidly frozen in liquid nitrogen and preserved at −80°C for Tissue homogenate kit analysis and 16S rDNA Gene Sequencing Analysis.

### Determination of Serum Biomarkers

According to the manufacturer’s commercially available kit instructions (Nanjing Jiancheng Bioengineering Institute, Nanjing, China), the activities of alanine aminotransferase (ALT), aspartate aminotransferase (AST), lactate dehydrogenase (LDH), Albumin (Alb) and gamma-glutamyl transferase (γ-GT) in serum were determined by an enzymatic colorimetric method (Wu et al., 2019).

### Assay of IL-6 and TNF-α in Liver

Weigh about 1 g of liver tissue, and add 9 times ice of normal saline, cut the tissue as much as possible with small ophthalmic scissors, use tissue homogenizer to grind into 10% tissue homogenate. The prepared homogenate was prepared with centrifuge at 4°C, 3,000 r/min, 15min centrifugation for supernatant. The levels of IL-6 and TNF-α in liver tissues were determined according to the enzyme-linked immunosorbent assay (ELISA) kit instructions (Shanghai Yaji Biological Co., Ltd., Shanghai, China).

### Pathological Assessment of Liver and Colon Tissue

The liver tissues and colon tissues were fixed in 4% paraformaldehyde. The tissues were subsequently dehydrated in a graded ethanol series (75–100%) and embedded in paraffin wax. The tissues were sectioned at 4 μm thickness, liver tissue was stained with hematoxylin and eosin (HE) and Masson, and colon tissue was stained with HE, digitally photographed by light microscopic at total magnifications of ×100.

### Determination of Fecal Microbial Composition by16S rDNA Gene Sequencing Analysis

Total Bacterial DNA was isolated from rat fecal samples by TIANamp Stool DNA Kit (TianGen Biotech Co., Ltd., BeiJing, China) and the quality of DNA extraction was detected by 1.2% agarose gel electrophoresis. Design primers based on conserved regions in sequences and add sample specific Barcode sequence. Then the 16S V3-V4 regions were PCR amplified. PCR amplified recovery products were quantified by fluorescence, and each sample was mixed according to the corresponding proportion according to the requirement of sequencing quantity of each sample according to the fluorescence quantitative results. The sequencing library was prepared using the TruSeq Nano DNA LT Library Prep Kit of Illumina company. Finally, high-throughput sequencing. All procedures, analysis of sequencing and the extracted DNA was subjected to Illumina Miseq PE250 platform. The content of the sequencing was commissioned to Shaanxi Irun Biotechnology Co., China.

### *In vivo* Anti-Inflammatory Effect Study of CGEA

Twenty-four healthy Kunming mice, weighing 25 ± 2 g, were provided by the Xinjiang medical university Experimental Animal center (Xinjiang China) [No. SCXK (Xin) 2016-0004], and all experimental procedures were approved by the Ethics Committee of First Affiliated Hospital, Shihezi University School of Medicine (Approval No. 2020-035-01).

After one week of adaptive feeding, the mice were randomly divided into four groups, namely Normal group, Model group, Treatment group, and Positive group, with six mice in each group. The mice were numbered and weighed, and the Treatment group was given 100 mg kg^−1^ CGEA by oral gavage, the Normal group and the Model group were given saline by oral gavage in equal amounts, and the Positive group was treated with 10 mg kg^−1^ dexamethasone sodium phosphate injection (Hubei Qianjiang Pharmaceutical Co., Ltd., Qianjiang, China) by intraperitoneal injection. All mice were treated twice daily for 7 days, and all mice were fasted but not water after the last dose on the seventh day. Except for the normal group, a single intraperitoneal injection of LPS at a dose of 15 mg kg^−1^ was administered to all groups on day 8 for the establishment of an acute inflammatory model in mice, and blood was obtained immediately after 3 h by removing the eyeballs.

### Biochemical Indexes Detection

After the blood was removed from the eyeball, it was left for 30 min, then centrifuged at 4°C, 3,000 r·min^−1^ for 20 min, and the serum was removed, and the content of IL-1β and IL-6 in the serum was measured in strict accordance with the instructions of ELISA kit (Shanghai Yaji Biological Co., Ltd., Shanghai, China).

### Determination of Liver and Spleen Index

After blood collection, cervical dislocation and execution of each group of mice, the spleen and thymus were removed intact, washed with saline, swabbed dry, weighed the mass, and calculated the spleen and thymus index.

Organ index (mg·g^−1^) = Organ mass (mg)/Body mass (g).

### Evaluation of CGEA in Promoting Probiotic Growth *in vitro*

Bifidobacterium (BBL) adolescentis [ATCC15703] and *Lactobacillus* acidophilus [ATCC4356] suspensions (10^7^ CFU mL^−1^) were transferred to BBL and DeMan-Rogosa-Sharpe (MRS) liquid medium containing 50, 25, 12.5, 6.25, 3.125, 1.56, 0.78, 0.39, 0.195, and 0.0975 mg mL^−1^ CGEA extracts, respectively, and the initial bacterial volume was adjusted to 10^6^ CFU mL^−1^. The same volume of bacterial suspension without CGEA was used as a normal control, and the wells containing only liquid medium were used as a reference control, and incubated for 12 h at 37°C under anaerobic conditions, and then the optical density value (OD value) of bacterial suspensions at 600 nm were measured with a microplate reader (Thermo Scientific, United States) to determine the optimal growth-promoting concentrations.

### The Growth Curve Method was Utilized to Observe the Bacterial Growth Status

The bacteria were treated with the same operation as above, and the optimal concentration of CGEA was added respectively. Under anaerobic conditions, the bacteria were incubated at 37°C for 24 h, and the OD value of the bacteria suspended at 600 nm wavelength was measured every 2 h with a microplate reader, and the bacterial growth curve was plotted with the measurement time and its corresponding OD value to evaluate the effect of CGEA on the growth process of the strain.

### Cell Culture and Processing

RAW 264.7 cells were donated by Xinjiang Uygur Autonomous Region Institute of Medicine, Maintain the cells in DMEM/High Glucose medium (HyClone, Logan, Utah, United States) supplemented with 10% v/v fetal bovine serum (FBS, Gibco, United States) and 1% penicillin-streptomycin solution (HyClone, Logan, Utah, United States), and put them into 25 cm^2^ culture flasks, and then place them in a constant temperature incubator (Thermo Fisher Scientific, United States) at 37°C and 5% CO_2_, Subculture every 2–3 days to maintain logarithmic growth. DMSO (Beijing Solarbio Technology Co., BeiJing, China) was used to prepare Lactucin solutions of different concentrations and added to the cell culture fluid. The proportion of DMSO in the cell culture fluid was 0.1% (v/v).

### Cell Viability

MTT (Beijing Solarbio Technology Co., BeiJing, China) was used to determine the effect of Lactucin on cell viability. Cells were propagated in a 96-well flat-bottom plate at a cell density of 1 × 10^5^ and combined for 16 h, the cells were counted using a hemocytometer. Then, the medium in the wells was discarded, and medium containing different concentrations of Lactucin was used to treat the cells for 24 h. At the end of incubation, each well was added with 10% MTT solution and the cells were cultured for another 4 h. Then carefully aspirate the culture solution, add 110 μL of formazan solution to each well, shake on a shaker at low speed for 10 min, and measure each wavelength at 490 nm by a microplate reader the absorbance (OD) value of the well.

### Determination of NO, IL-6 and TNF-α Production

Cells were implanted in 96-well flat-bottom plates at a cell density of 1 × 10^5^ for 16 h, and then the culture solution was discarded. The cells in the normal group and the model group were added with DMEM/High Glucose medium, and the drug intervention group was added with low (12.5 μmol L^−1^), medium (25 μmol L^−1^) and high (50 μmol L^−1^) doses of Lactucin medium to pre-protect the cells for 30 min. Except for the normal group, LPS (Solarbio Technology Co., BeiJing, China) (1 μg mL^−1^) was added to each well, and the culture was continued for 24 h. To measure NO, mix 100 μL of cell supernatant with an equal volume of Griess reagent (Hu et al., 2016a), measure the absorbance of each well at a wavelength of 550 nm using a microplate reader within 5 min, and calculate the content of NO in the cell culture solution. The levels of IL-6 and TNF-α (Shanghai Yaji Biological Co., Ltd., Shanghai, China) in the culture medium were measured using Elisa kit.

### RNA Extraction and Quantitative Real Time-PCR Analysis

Cells were implanted in 6-well plate at a density of 1 × 10^6^ and incubated for 16 h. Then, the drug intervention group was added with low (12.5 μmol L^−1^), medium (25 μmol L^−1^) and high doses (50 μmol L^−1^) of Lactucin medium to pre-protect the cells for 30 min. Except for the normal group, LPS (1 μg mL^−1^) was added to each well, and the culture was continued for 6 h, and then, according to the manufacturer’s instructions, total cell RNA was extracted using Trizol reagent (Invitrogen, Carlsbad, CA, United States). First-strand cDNA was synthesized from the total RNA (1 μg) was used the PrimeScript™ RT reagent Kit with gDNA Eraser (TaKaRa Corporation, Japan). Then use the LightCycler 480 PCR fluorescence quantifier (Roche, Germany) to amplify the target gene according to the method reported previously. The conditions for qPCR were 95°C for 10 min followed by 45 cycles at 95°C for 15 s, 57°C for 15 s, and 72°C for 15 s, with a final extension at 72°C for 10 min. GAPDH was used as the house-keeping genes, and the relative expression change factor of each target gene was calculated by 2^-△△CT^ method (Hu et al., 2016b). Table 1 lists the oligonucleotide primers of the target gene.

**TABLE 1:** Primer sequences.

Gene	Forward primer 5′-3′	Reverse primer 5′-3′	Amplified fragment
GAPDH	AGG​AGA​GTG​TTT​CCT​CGT​CC	TGA​GGT​CAA​TGA​AGG​GGT​CG	145 bp
IL-6	CCG​GAG​AGG​AGA​CTT​CAC​AG	TCC​ACG​ATT​TCC​CAG​AGA​AC	121 bp
IL-1β	TGC​AGA​GTT​CCC​CAA​CTG​GTA​CA	GTG​CTG​CCT​AAT​GTC​CCC​TTG	102 bp
TNF-α	TCA​GCC​TCT​TCT​CAT​TCC​TG	TGA​AGA​GAA​CCT​GGG​AGT​AG	136 bp
iNOS	CCC​TTC​CGA​AGT​TTC​TGG​CAG​CAG	GGC​TGT​CAG​AGC​CTC​GTG​GCT​TTG​G	108 bp
COX-2	CAC​TAC​ATC​CTG​ACC​CAC​TT	ATG​CTC​CTG​CTT​GAG​TAT​GT	258 bp

### Western Blot Analysis

A total of 1 × 10^6^ cells plated in a 6-well plate for 16 h and then exposed to Lactucin for 30 min before stimulation with LPS for indicated time. After washing the cells twice with cold PBS, the cells were scraped to collect the cells, 100 μL of radio immunoprecipitation assay (RIPA) lysis buffer (CoWin Biosciences Co., BeiJing, China) was added, shaken thoroughly and placed in a refrigerator at 4°C, lyzed for 30 min, and then centrifuged at 4°C and 12,000 r/min for 10 min, extracted the total protein of the cell, and used the bicinchoninic acid (BCA) protein concentration determination kit (Solarbio Life Sciences, BeiJing, China) for concentration determination. Sodium dodecyl sulfate-polyacrylamide gel electrophoresis (SDS-PAGE) was used to separate the proteins (50 μg) in each sample and transferred onto polyvinylidene fluoride (PVDF, Millipore, Billerica, MA, United States) membranes. The PVDF membranes were then incubated overnight at 4 °C with primary antibodies including iNOS antibody (1:1,000, Cell Signaling Technology, United States), COX-2 antibody (1:1,000, Bioworld, NanJing China), P-Akt antibody (1:1,000), Akt antibody (1:2000) all from (Boster Biological technology, WuHan, China), p38 antibody (1:2000), P-p38 antibody (1:2000), ERK1/2 antibody (1: 2000), P-ERK1/2 antibody (1:2000), JNK antibody (1:2000), P-JNK antibody (1:2000), GAPDH antibody (1:2000), Actin antibody (1:2000), all from (Beyotime Biotechnology, ShangHai, China). The membranes were then incubated for 1 h with horseradish peroxidase (HRP)-conjugated goat anti-rabbit/mouse IgG antibody (1:5,000, Zhongshan Jinqiao Biotechnology Co., Beijing, China), and the immunoreactive proteins were visualized using the enhanced chemiluminescence reagent (ECL, Solarbio Technology Co., Beijing, China). The images were obtained using ChemiDoc™ Omnimaging System (Bio-Rad, Hercules, CA, United States) and developed using ImageJ (NIH, United States) Software for quantification.

### Statistical Analysis

Each group of experiments was independently repeated 3 times, and the data were expressed as mean ± standard deviation (SD). All date were analyzed with Graph Pad Prism 6.0 software (California, United States). One-way analysis of variance (ANOVA) was used to compare differences among multiple groups, and unpaired Student’s t-test was used to analyze the significance between two groups. Values of *p* < 0.05 was considered statistically significant.

## Results

### HPLC Analysis

**FIGURE 1:**
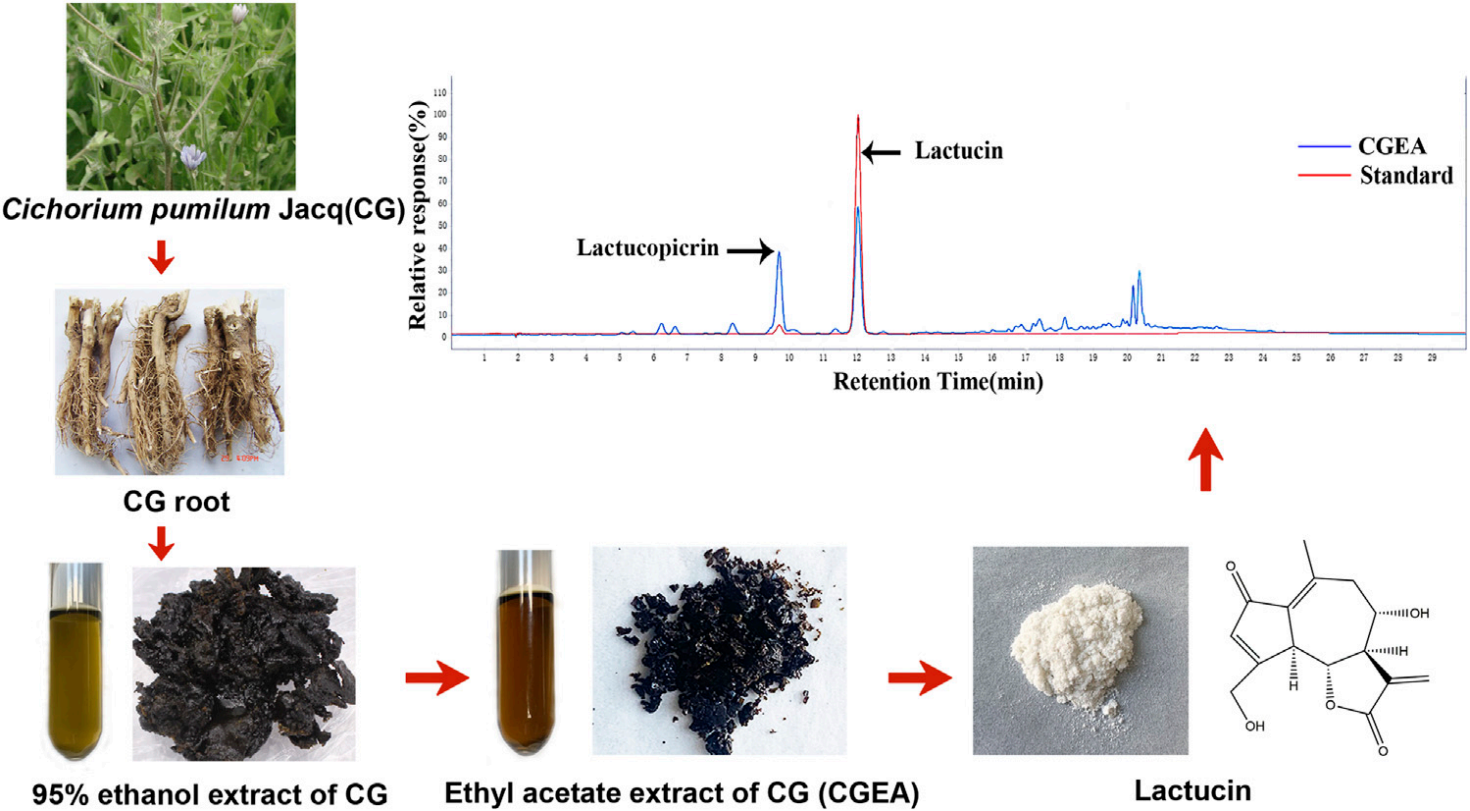
Preparation of CGEA and Lactucin and their analysis by HPLC, HPLC chromatogram of CGEA at 275 nm, peaks is Lactucin.

By comparing the retention times of CGEA and Lactucin in the chromatograms, we found that the major component and the observed peak at 275 nm were identified as Lactucin (Figure 1) at a concentration of 6%.

### CGEA Attenuates Liver Fibrosis in Rats

**FIGURE 2:**
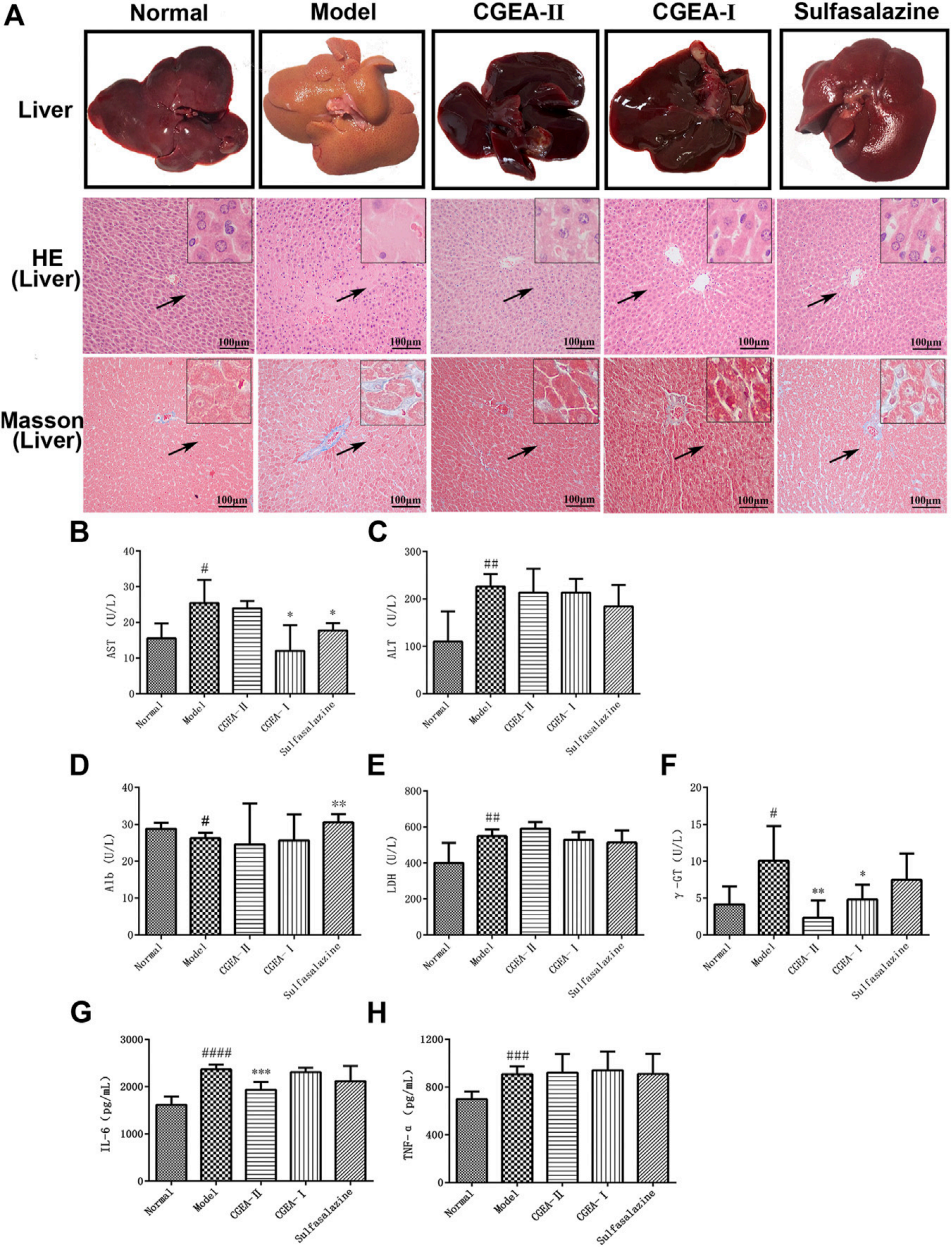
CGEA reduces liver index and inflammation index of liver fibrosis rats. **(A)** Typical images of representative liver pathology for HE staining and Masson staining. **(B–F)** Effects of CGEA on serum AST, ALT, Alb, LDH, and *γ*-GT activities. **(G–H)** Statistic analysis of liver inflammation scoring (IL-6, TNF-α). Data are shown as mean ± SD in each group (*p < 0.05, **p < 0.01, ***p < 0.001 with the Model Group; #*p* < 0.05, ##*p* < 0.01, ###*p* < 0.001, ####*p* < 0.0001 with the Normal Group; *n =* 6). Abbreviations: Normal, Healthy rats; Model, Rats with enteritis and liver fibrosis; CGEA-Ⅱ, CGEA low-dose treatment group; CGEA-Ⅰ, CGEA high-dose treatment group; AST, aspartate transaminase; ALT, alanine transaminase; Alb, albumin; LDH, lactate dehydrogenase; *γ*-GT, *γ*-glutamyl transpeptidase.

In the process of establishing the animal model, the rats showed thin feces, blood feces, which showed obvious characteristics of colitis. From the fourth week, we used orbital blood collection to determine AST and ALT indicators in some of the rats, and at 6 weeks, AST and ALT in the rats’ serum began to appear abnormal compared with the normal group, indicating that the rats began to show liver damage. Our previous experimental results have shown that liver damage lasting more than 6 weeks can cause liver fibrosis (Qin et al., 2019a; Qin et al., 2019b). Therefore, we continued to give rats enemas with TNBS for up to 12 weeks to promote the formation of liver fibrosis in rats. The results showed that no distinct edema lesions or abnormal color was observed in liver tissue of the control group. In contrast, the model group significantly resulted in ischemia and abnormal color of rat liver tissue, however, both CGEA and Sulfasalazine significantly improved the liver congestion function and normalized the color of the liver (Figure 2A). The AST, ALB and *γ*-GT in serum were significantly increased in the model group compared with those of normal group (*p* < 0.05) and ALT, LDH was significantly increased (*p* < 0.01) (Figures 2B–F). CGEA-Ⅰ can significantly reduce the levels of AST (Figure 2B) and *γ*-GT (Figure 2F) (*p* < 0.05), CGEA-Ⅱ also significantly reduced the level of *γ*-GT (*p* < 0.01) (Figure 2F). In conclusion, we successfully created a new liver injury-hepatic fibrosis model using TNBS-ethanol enemas, and CGEA mitigated liver fibrosis in this model.

### CGEA Significantly Improves Histopathological Changes in Liver Fibrosis in Rats

The results of HE staining showed that, compared with those of the normal group, the liver lobules of the model group were blurred in outline, the structure was obviously damaged, the central veins were dilated, the arrangement of the hepatic cord was disordered, and there was a large number of inflammatory cells infiltrating the hepatic lobules and confluent area (Figure 2A). Masson’s staining results showed that collagen fibrous tissue proliferation and collagen deposition increased around the central vein and in the confluent area of the liver of the model group rats, forming obvious fibrous intervals with typical histological features of liver fibrosis. Compared with those of the normal group, collagen fibers were mainly distributed in the liver interstitium in the CGEA group and were significantly reduced, especially in the CGEA-Ⅰ group (Figure 2A). In conclusion, the rats in this experiment have typical liver fibrosis characteristics, and CGEA can significantly reduce the collagen fibrillar content in the liver, with significant anti-fibrotic activity.

### CGEA Reduces Inflammatory Factor Levels in Rat Liver

In order to verify that the effect of CGEA on liver fibrosis in experimental rats is achieved by reducing liver inflammation, we determined the levels of IL-6 and TNF-α in rat liver tissue using the ELISA method. TNF-α and IL-6, are important inflammatory response factors in the organism that promotes fibrosis formation by inducing inflammatory responses in the liver. The results of this study showed that CGEA-II group could significantly reduce the level of IL-6 (*p* < 0.001) (Figure 2G), the effect of CGEA on the level of TNF-α was not significant (*p* > 0.05) (Figure 2H).

### CGEA Improves Colonic Tissue Changes in Rats With Colitis

**FIGURE 3:**
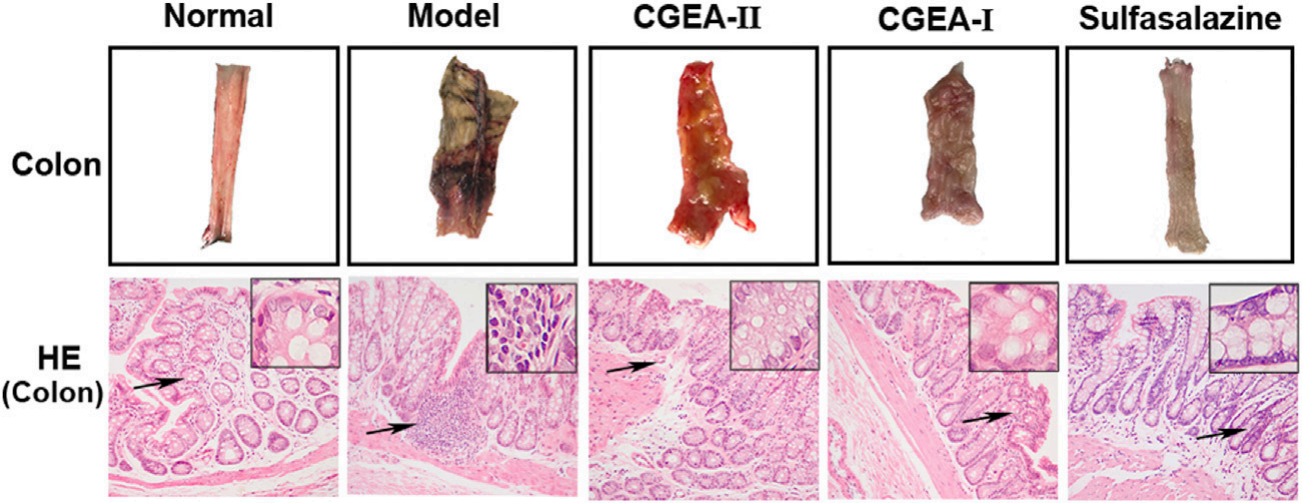
CGEA restores the colonic barrier function of rats with liver fibrosis. Representative colon tissue images and HE-stained images(Normal: Healthy rats, Model: Rats with enteritis and liver fibrosis, CGEA-Ⅱ: CGEA low-dose treatment group, CGEA-Ⅰ: CGEA high-dose treatment group.)

The results showed that the colon in the control group had no obvious edema and no ulceration, while the colonic tissue in the model group showed obvious ulceration and tissue necrosis, and the CGEA intervention could significantly improve the lesions in the colonic tissue (Figure 3). Similarly, the results of HE staining showed that there was no damage to the colon tissue of the normal group of rats, and the glandular structure in the colon was intact. In the model group, the mucosal layer of the colonic tissue was severely damaged, with loss of microvilli and glandular fragmentation. The colon of the treated group had a large amount of granulation tissue proliferation and epithelial regeneration, especially the CGEA-I group had the best repair effect (Figure 3). It means that CGEA treatment can significantly improve the damage of colon tissue and reduce the inflammation of colon.

### CGEA Alters the Composition of the Microbiota in the Gut of Rats With Hepatic Fibrosis

**FIGURE 4:**
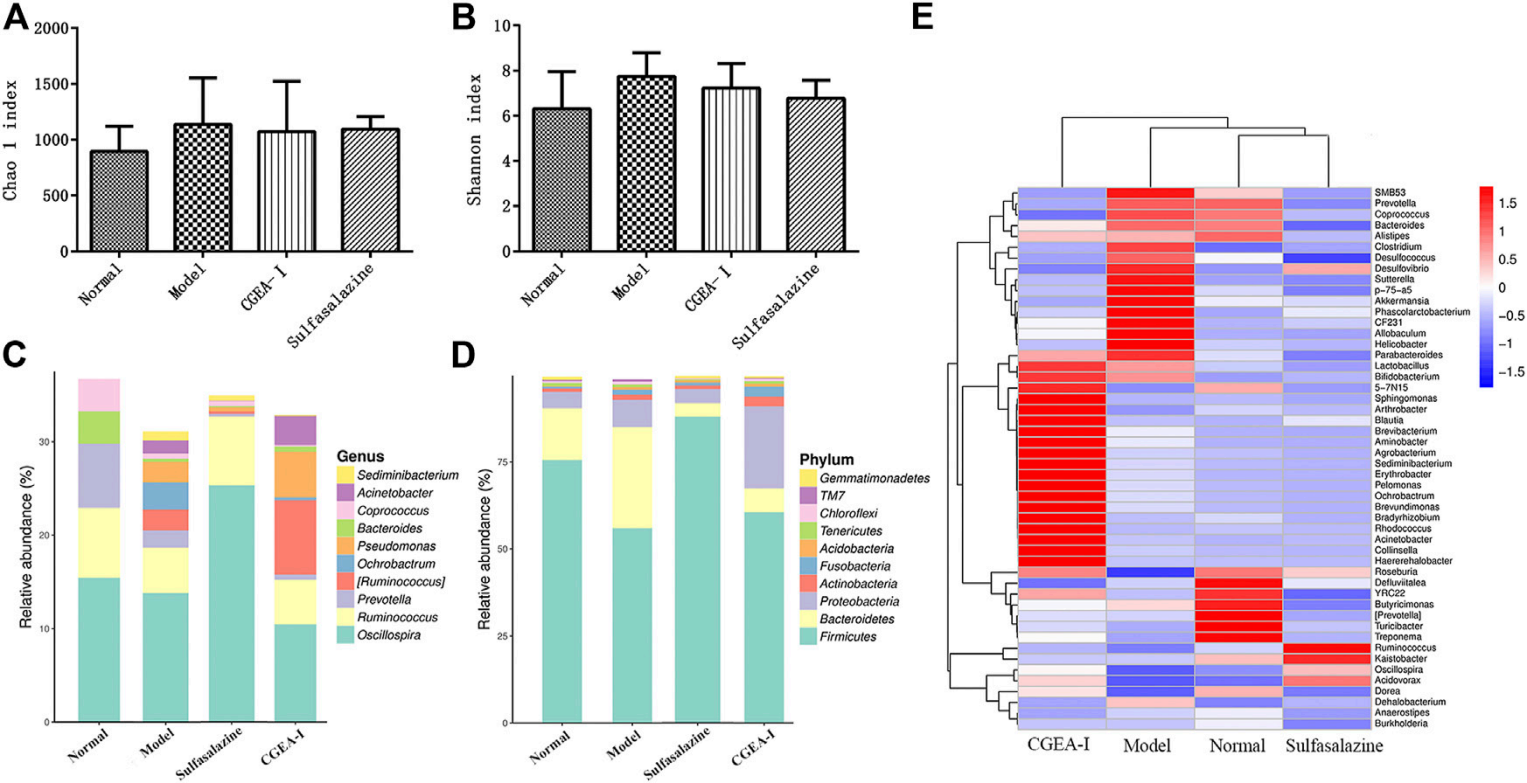
CGEA reverses the changes of intestinal microbes. **(A–B)** Effects of CGEA on the observed species Chao one index and Shannon index in fecal microbiota after long-term TNBS exposure, respectively. **(C)** CGEA significantly ameliorated the relative abundance changes of fecal microbial composition at the genus level due to TNBS intake. **(D)** CGEA dramatically modified TNBS-induced relative abundance alterations of fecal microbial composition at the phylum level. **(F)** Heat map analysis of the gut microbiota. Data are shown as mean ± SD from three independent experiments (Normal: Healthy rats, Model: Rats with enteritis and liver fibrosis, CGEA-Ⅰ: CGEA high-dose treatment group).

The Chao1 index and Simpson index represent the richness and diversity of microbes in the gut. Compared with those of the normal group, the enrichment of gut microorganisms in the TNBS group increased slightly and the diversity decreased, while the CGEA and positive drug groups corrected this change, but there was no significant difference among all groups (*p* > 0.05) (Figures 4A,B). In order to observe specific changes in microorganisms in the gut, we genetically sequenced microorganisms in intestinal feces by 16S rDNA, and it is noteworthy that the TNBS group and the administration intervention group caused changes in bacterial communities at the level of phylum and genus, and at the taxonomic level of phylum, Proteobacteria, Firmicutes and Bacteroidetes represent the majority of the total sequence of bacteria. Compared with those of the normal group, the abundance of Firmicutes in the intestines of rats in the TNBS group was significantly reduced and the abundance of Bacteroidetes was significantly increased, while the CGEA group and the positive drug group improved this situation by increasing the abundance of Firmicutes, Proteobacteria and Acidobacteria and reducing the abundance of Abundance of Bacteroidetes. (Figure 4D); at the level of genus, compared with those of the normal group, after TNBS modeling, the abundance of Prevotella in the intestines of rats was reduced, and the abundance of *Pseudomonas* and Ochrobactrum was increased, and the CGEA intervention could reduce the abundance of Ochrobactrum and increase the abundance of Ruminococcus and *Acinetobacter*, making the intestinal flora more to the normal level (Figure 4C).

### CGEA Reduces the Level of Inflammation and Improves the Organ Index in Mice

**FIGURE 5:**
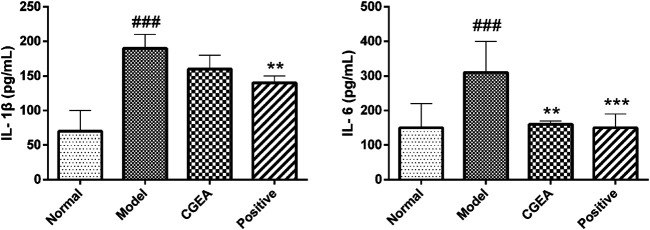
CGEA reduces the serum levels of IL-1β and IL-6 in inflamed mice. Data are shown as mean ± SD in each group (**p < 0.01, ***p < 0.001 with the Model Group; ###*p* < 0.001 with the Normal Group; *n =* 6). Abbreviations: Normal, Healthy mice; Model, LPS-induced inflammation model mice; CGEA, 100 mg ml^−1^ dose treatment group; Positive, dexamethasone sodium phosphate injection treatment group.

Compared with the normal group, IL-1β and IL-6 in the serum of mice in the model group were highly significantly elevated (*p* < 0.001). Compared with the model group, CGEA significantly reduced the level of IL-6 in the serum of mice (*p* < 0.01). Although CGEA could not significantly reduce the level of IL-1β, the results could indicate that CGEA had a decreasing effect on IL-1β (Figure 5). Similarly, CGEA also reduced the spleen index and thymus index in mice (Table 2).

**TABLE 2:** Effect of CGEA on spleen index and thymus index in mice with LPS-induced acute inflammation (*x* ± *s*, *n =* 6).

Group	Dosage (mg·kg^−1^)	Spleen index (mg·g^−1^)	Thymus index (mg·g^−1^)
Normal	—	3.08 ± 0.49	1.37 ± 0.39
Model	—	4.00 ± 0.85	1.66 ± 0.96
CGEA	100	3.00 ± 0.46	1.57 ± 0.51
Positive	10	2.62 ± 0.87*	1.39 ± 0.16

* P < 0.05 with the Model Group.

### CGEA Promotes the Growth of Bifidobacterium Adolescentis

**FIGURE 6:**
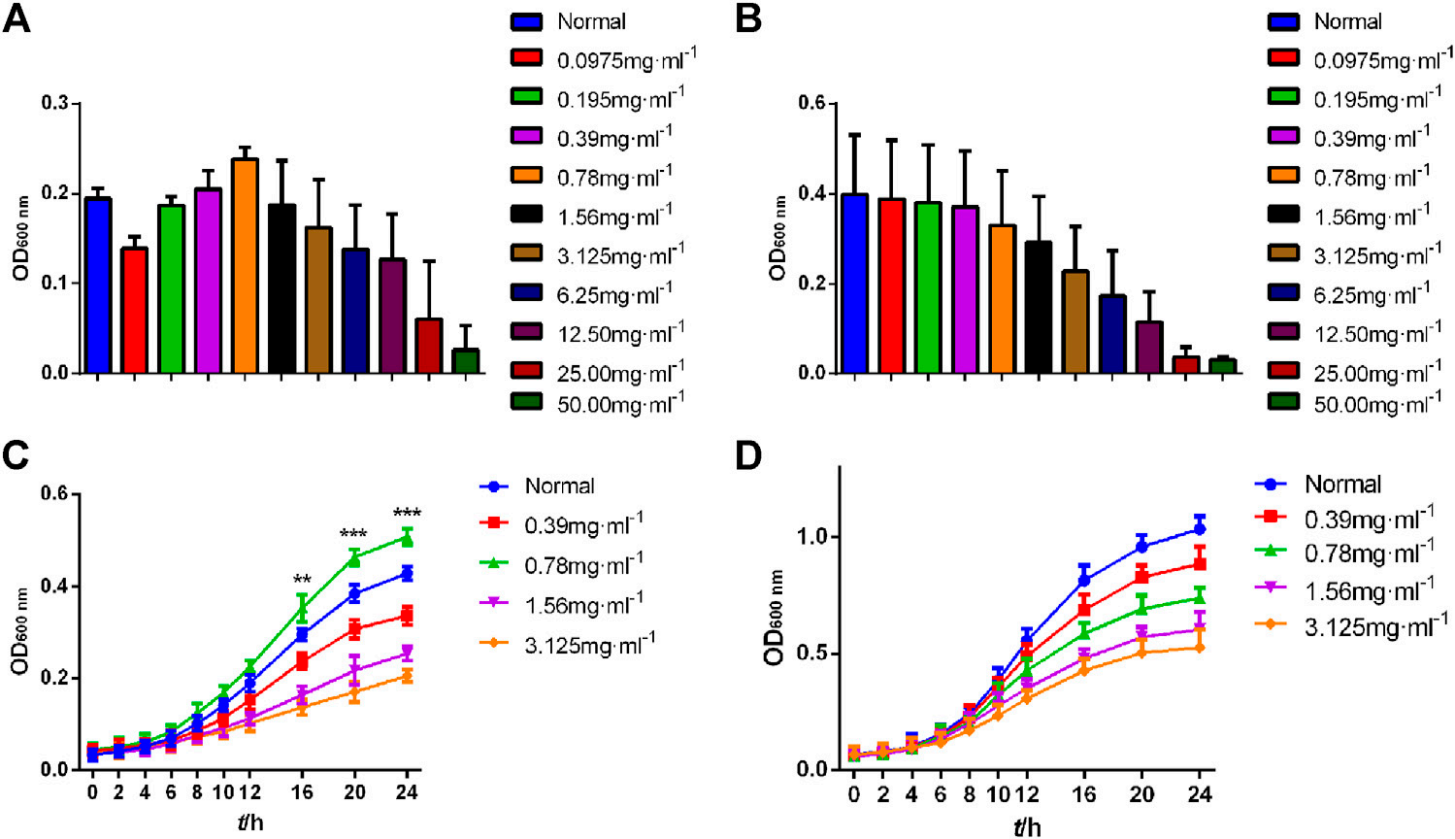
Growth curve of Bifidobacterium adolescentis and *Lactobacillus* acidophilus. Data are shown as mean ± SD in each group (**p < 0.01, ***p < 0.001 with the Normal Group; *n = 3*). **(A, C)** Bifidobacterium adolescentis. **(B, D)** *Lactobacillus* acidophilus. Abbreviations: Normal, The bacterial suspension without CGEA.

Compared with the Normal group, high concentrations of CGEA significantly inhibited the growth of Bifidobacterium adolescentis and *Lactobacillus acidophilus*, and as the concentration decreased, CGEA showed some microbial growth promoting effects, especially at a concentration of 0.78 mg mL^−1^, the growth promoting effect of CGEA on Bifidobacterium adolescentis was the most obvious (Figure 6A). Further, the growth curve results showed that with the decrease of CGEA concentration, CGEA at 0.78 mg mL^−1^ concentration could significantly promote the growth of Bifidobacterium adolescentis after 16 h of co-interaction (*p* < 0.01), and when the concentration continued to decrease, this promotion effect started to weaken again (Figure 6C); the effect of CGEA on the growth promoting effect of CGEA on *Lactobacillus acidophilus* was not significant, but the toxic effect of low concentration of CGEA on *Lactobacillus acidophilus* was also very weak and basically did not affect the normal growth of *Lactobacillus acidophilus* (Figure 6D).

### Lactucin Significantly Reduces LPS-Induced Levels of Inflammatory Mediator Production in RAW264.7 Cells

**FIGURE 7:**
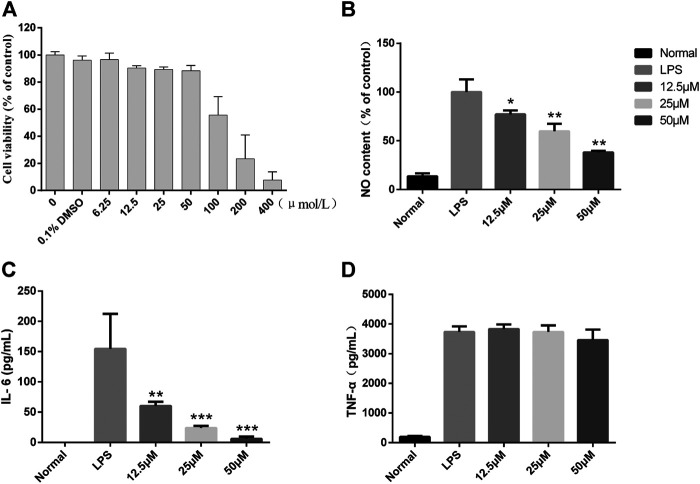
*In vitro* anti-inflammatory effect of Lactucin in LPS-activated RAW 264.7 cells. **(A)** The cell viability of RAW264.7 cells were assessed by MTS assay. **(B)** The concentrations of NO were measured using the Griess reaction, and Lactucin significantly inhibited NO production. **(C, D)** The levels of IL-6 and TNF-α in the culture supernatants were determined by ELISAs, and Lactucin significantly inhibited IL-6 production. Data are shown as mean ± SD in each group (*p < 0.05, **p < 0.01, ***p < 0.001 with the LPS Group, *n =* 3. Normal Group: RAW264.7 cells without LPS activation).

To further clarify the potent anti-inflammatory activity of CGEA, we investigated the mechanism of anti-inflammatory action of its main compound, Lactucin, *in vitro*. First, the toxic effect of Lactucin on RAW264.7 cells was determined by MTT, and the results showed that when Lactucin was 50 μmol L^−1^, the cell viability was recovered 88.08%, which can be considered as no significant cytotoxicity. Similarly, DMSO at a final concentration of 0.1% was not cytotoxic, whereas 100, 200, and 400 μmol L^−1^ all showed significant cytotoxicity with cell survival rates of 55.64, 23.40, and 7.66%, respectively (Figure 7A); therefore, subsequent experiments were performed using Lactucin at non-cytotoxic concentrations of 12.5, 25, and 50 μmol L^−1^, providing a basis for the variation in the experimental data not due to cytotoxicity. The results showed that 50 μmol L^−1^, 25 μmol L^−1^ Lactucin could significantly inhibit the level of NO production in RAW264.7 cells (*p* < 0.01), and 12.5 μmol L^−1^ Lactucin could also significantly inhibit NO production (*p* < 0.05) (Figure 7B); 50 μmol L^−1^, 25 μmol L^−1^ Lactucin could significantly inhibit IL-6 production (*p* < 0.001), 12.5 μmol L^−1^ Lactucin could also significantly inhibit IL-6 production (*p* < 0.01) (Figure 7C); and the inhibitory effect of Lactucin on TNF-α was not significant (*p* > 0.05) (Figure 7D).

### Lactucin Significantly Inhibits LPS-Induced Inflammatory mRNA Expression Levels in RAW264.7 Cells

**FIGURE 8:**
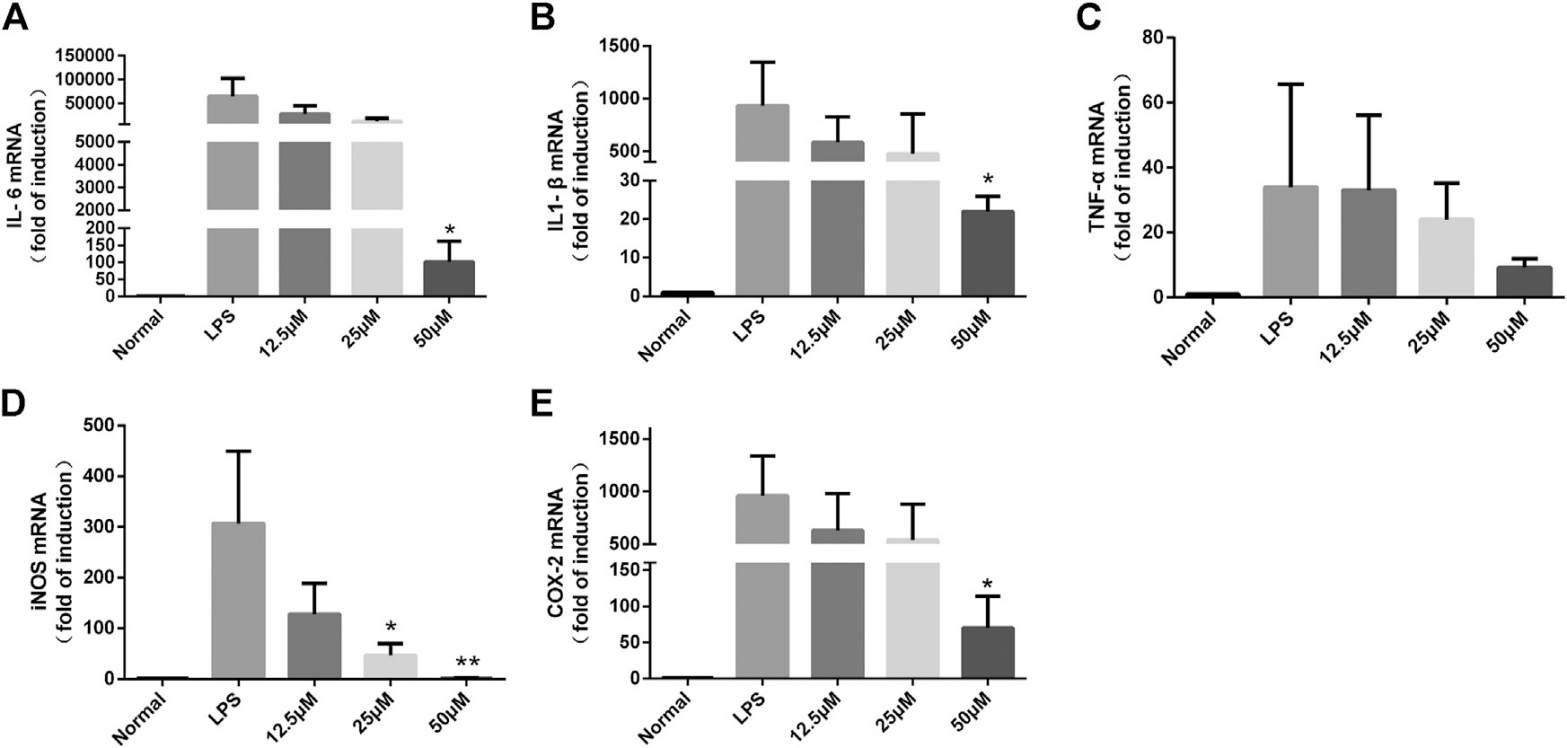
The effect of Lactucin on mRNA expression of a set of inflammatory genes. and Lactucin significantly inhibited IL-6, IL1-β, iNOS, and COX-2 mRNA expression. Data are shown as mean ± SD for each group (*p < 0.05, **p < 0.01 with the LPS Group, *n =* 3. Normal Group: RAW264.7 cells without LPS activation).

It is well known that iNOS is a key enzyme for cellular production of large amounts of NO (Cassini-Vieira et al., 2015), and in addition, COX-2 is an inflammatory gene that catalyzes the production of PGE_2_ and is involved in promoting the inflammatory response process (He et al., 2020). To further investigate whether the inhibitory effects of lactucin on NO, PGE_2_, IL-6, TNF-α, and IL-1β are related to the genes responsible for regulation, we determined the effects of lactucin on the expression of mRNA with relevant targets. The results showed that the mRNA levels of the target genes were undetectable in the unstimulated RAW264.7 cells. After LPS stimulation, the mRNA expression of iNOS, COX-2, IL-6, IL-1β and TNF-α was significantly increased. Lactucin significantly inhibited the mRNA expression of these genes in a concentration-dependent manner, in which 50 μmol L^−1^ of Lactucin significantly inhibited the mRNA expression of IL-6, IL-1β and COX-2 (*p* < 0.05) (Figure 8A,B,E) and extremely significantly inhibited the mRNA expression of iNOS (*p* < 0.01), and 25 μmol L^−1^ of Lactucin had a significant inhibitory effect on the mRNA expression of iNOS (*p* < 0.05) (Figure 8D), Lactucin had an inhibitory effect on TNF-α mRNA expression, but there was no significant difference (*p* > 0.05) (Figure 8C). These results suggest that the inhibition of inflammatory mediators in LPS-stimulated macrophages by Lactucin can be explained by the inhibition of the mRNA expression achieved in the production of inflammatory mediators.

### Lactucin Significantly Inhibits iNOS, COX-2 Protein Expression

**FIGURE 9:**
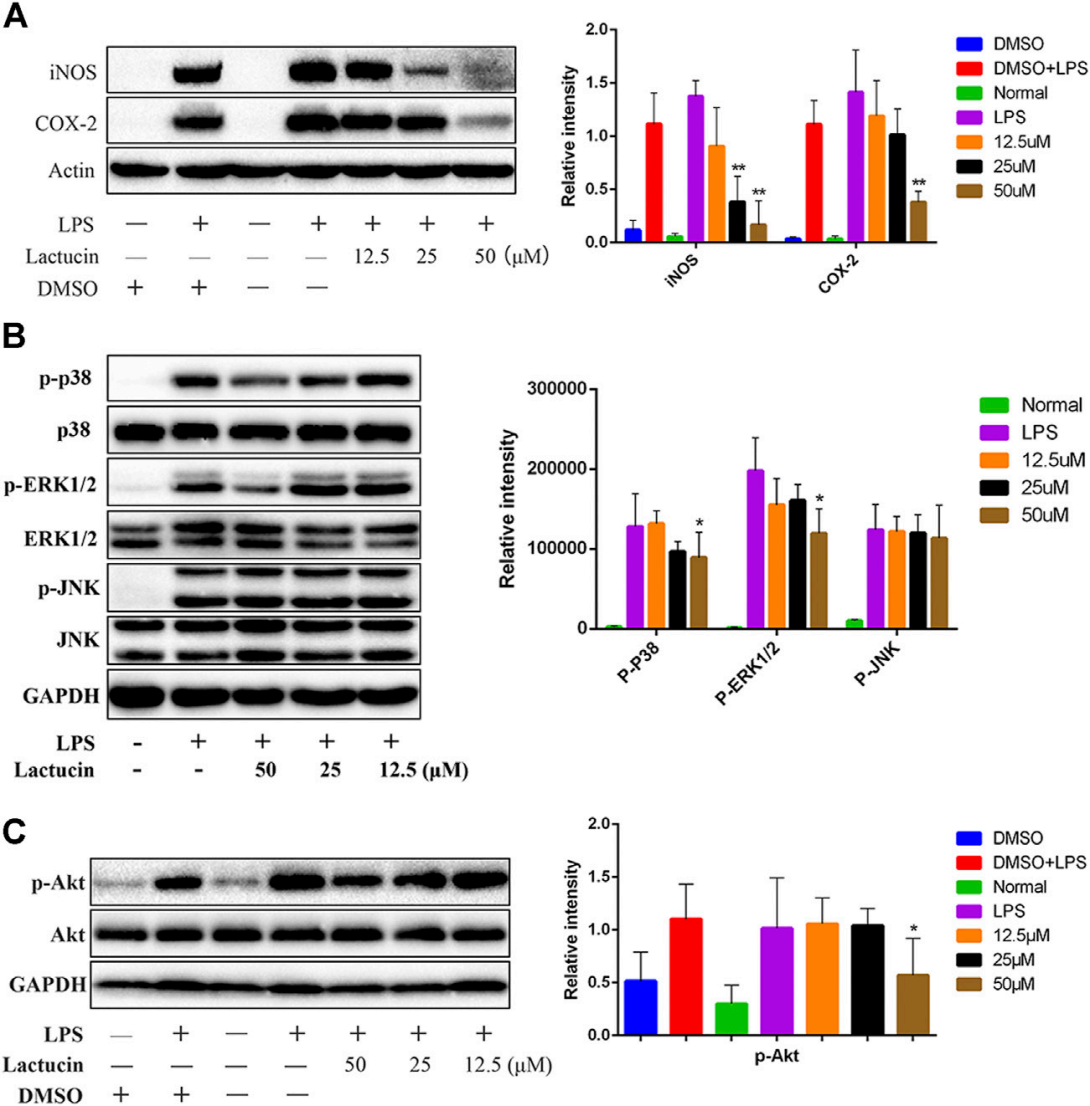
Effect of Lactucin on the activation of signaling pathways. **(A)** The whole-cell lysates were extracted for immunoblotting to determine the level of iNOS, COX-2. **(B, C)** The whole-cell lysates were extracted for immunoblotting to determine the levels of phospho- or total MAPKs (ERK, p38, and JNK) and AKT identified based on their antibodies. Data are shown as mean ± SD for each group (*p < 0.05 with the LPS Group, *n =* 3. Normal Group: RAW264.7 cells without LPS activation).

To further investigate whether the inhibitory effect of Lactucin on inflammatory mediators is related to its associated proteins, Western Blot was used to determine protein expression of iNOS and COX-2. The results showed that after 12 h of LPS stimulation, iNOS and COX-2 proteins were highly expressed in the cells, whereas iNOS and COX-2 proteins were hardly expressed in normal cells, 50 μmol L^−1^ of lactucin could extremely significantly inhibit iNOS and COX-2 protein expression (*p* < 0.01), and 25 μmol L^−1^ of lactucin could also extremely significantly inhibit iNOS protein expression (*p* < 0.01), in addition, it is evident that 0.1% of DMSO had no effect on protein expression (Figure 9A).

### Lactucin Significantly Inhibits AKT, MAPKs Protein Phosphorylation

Dysregulation of MAPK signaling pathway is one of the key factors that induce inflammation. Inhibition of MAPK signaling pathway can ameliorate many inflammatory diseases (Gong et al., 2014). At the same time, inhibition of Akt protein phosphorylation also prevents NF-κB p50 subunit movement toward the cell nucleus, which in turn inhibits inflammatory factor production (Lai et al., 2015). The results showed that the phosphorylation of MAPKs and AKT proteins could be induced by LPS stimulation for 30 min 50 μmol L^−1^ of Lactucin significantly reduced the phosphorylation of p38, ERK1/2 (Figure 9B) and AKT proteins (*p* < 0.05) (Figure 9C), had no significant effect on the phosphorylation of JNK proteins, and almost no effect on the non-phosphorylated AKT, p38, ERK1/2, and JNK protein content.

## Discussion

In this study, we demonstrated that CGEA significantly ameliorated liver fibrosis induced by chronic colitis in rats. Promoting the growth of bifidobacteria. Interestingly, we found that CGEA could alter the composition of microorganisms in the rat intestine, and the gut-liver axis has been reported to be closely associated with liver fibrosis, however, intestinal microbes and intestinal barrier are two key factors associated with liver fibrosis (Tripathi et al., 2018). Our results found that persistent colitis disturbed microbial populations in the rat gut, as evidenced by up-regulating of the abundance of Bacteroidetes and down-regulating the abundance of Firmicutes in the intestine, which is consistent with a report by Frank (Frank et al., 2011), and the Bacteroidetes and Firmicutes, as the two main dominant groups in the intestine, participated in the metabolism and absorption of nutrients in the body. Firmicutes can help the body absorb more energy from food (Ley et al., 2006), thus increasing the body’s resistance, while patients with liver fibrosis have abnormal liver function and generally weak immunity. CGEA may improve the body’s immunity against liver fibrosis by reconfiguring microbial populations in the intestine.

On the other hand, when intestinal inflammation persists, the intestinal villi become sparse and damaged, the intestinal barrier function is disrupted, and the metabolites of microorganisms in the gut with harmful flora enter the liver with the enterohepatic axis, stimulating inflammation in the liver. Our results show that increased expression of inflammatory factors in the liver of rats with colitis reveals that functionally impaired intestinal microbes can stimulate intrahepatic inflammation and enter the enterohepatic circulation through the damaged intestinal barrier, exacerbating the development of liver fibrosis. The metabolism of short-chain fatty acids (SCFAs) produced by the Ruminococcus is one of the richest families of Fusobacteria under the Firmicutes (Sagheddu et al., 2016), which enhances the protective effect of the intestinal barrier (Kang et al., 2017; Porras et al., 2017). Therefore, we speculate that the protective effect of CGEA on the rat intestinal barrier may be related to its promotion of the growth of Ruminococcus, however, its specific mechanism of action needs to be further investigated.

Almost all liver diseases are accompanied by inflammation (Bai et al., 2017), and sustained inflammatory stimuli lead to substantial loss of hepatocytes and loss of liver function (Wu et al., 2019), thus reducing the production of inflammatory factors is important to reduce liver injury and fibrosis (O'Reilly et al., 2014; Seki and Schwable, 2015). IL-6 is a proinflammatory cytokine produced after immune activation that can exacerbate chronic inflammation (Cha et al., 2015). And our results table that CGEA significantly reduced serum levels of IL-6 both in acutely inflamed mice and in rats with liver fibrosis. In conclusion, our results suggest that CGEA can reduce the expression of inflammatory factors not only directly, but also indirectly by regulating microorganisms and improving the circulation of the enterohepatic axis.

Earlier experiments demonstrated the most significant anti-inflammatory activity of Lactucin in CGEA (Dang et al., 2019). In the present experiments we further determined the content of Lactucin and the HPLC results showed that the content of Lactucin in CGEA was 6%.Lactucin being the major compound in CGEA, we predict that the anti-inflammatory activity exhibited by CGEA is mainly due to the anti-inflammatory effect exerted by Lactucin. It is well known that dysregulation of MAPK signaling pathway is a key factor in triggering inflammation (Jeong et al., 2014; Duan et al., 2017). Moreover, MAPKs and PI3K/Akt signaling pathways can directly promote DNA binding activity, leading to the up-regulation of iNOS and TNF-α mRNA expression in RAW 264.7 cells (Hu et al., 2016a). The results showed that Lactucin could significantly inhibit LPS-induced iNOS and COX-2 mRNA expression and protein expression in RAW264.7 cells, which is consistent with the findings of Lee et al. (Lee et al., 2010). This suggests to us that Lactucin exert anti-inflammatory effects by suppressing the phosphorylation of ERK1/2 and p38 signaling pathways, which lead to the inhibition of NO production in RAW 264.7 cells.

PI3K/Akt is a serine/threonine-specific protein kinase that plays a key role in many cell growth processes and is closely related to the MAPK signaling pathway, which can both activate and inhibit each other (Meng et al., 2009; Aksamitiene et al., 2012). Our results indicate that Lactucin can significantly inhibit the mRNA expression of IL-6 and IL-1β in RAW264.7 cells and significantly inhibit the phosphorylation of AKT protein. In conclusion, the results here indicate that Lactucin is anti-inflammatory by inhibiting the activation of the MAPK-AKT signaling pathway in RAW264.7 cells, thereby inhibiting the production of inflammatory factors such as NO and IL-6.

## Conclusion

**FIGURE 10:**
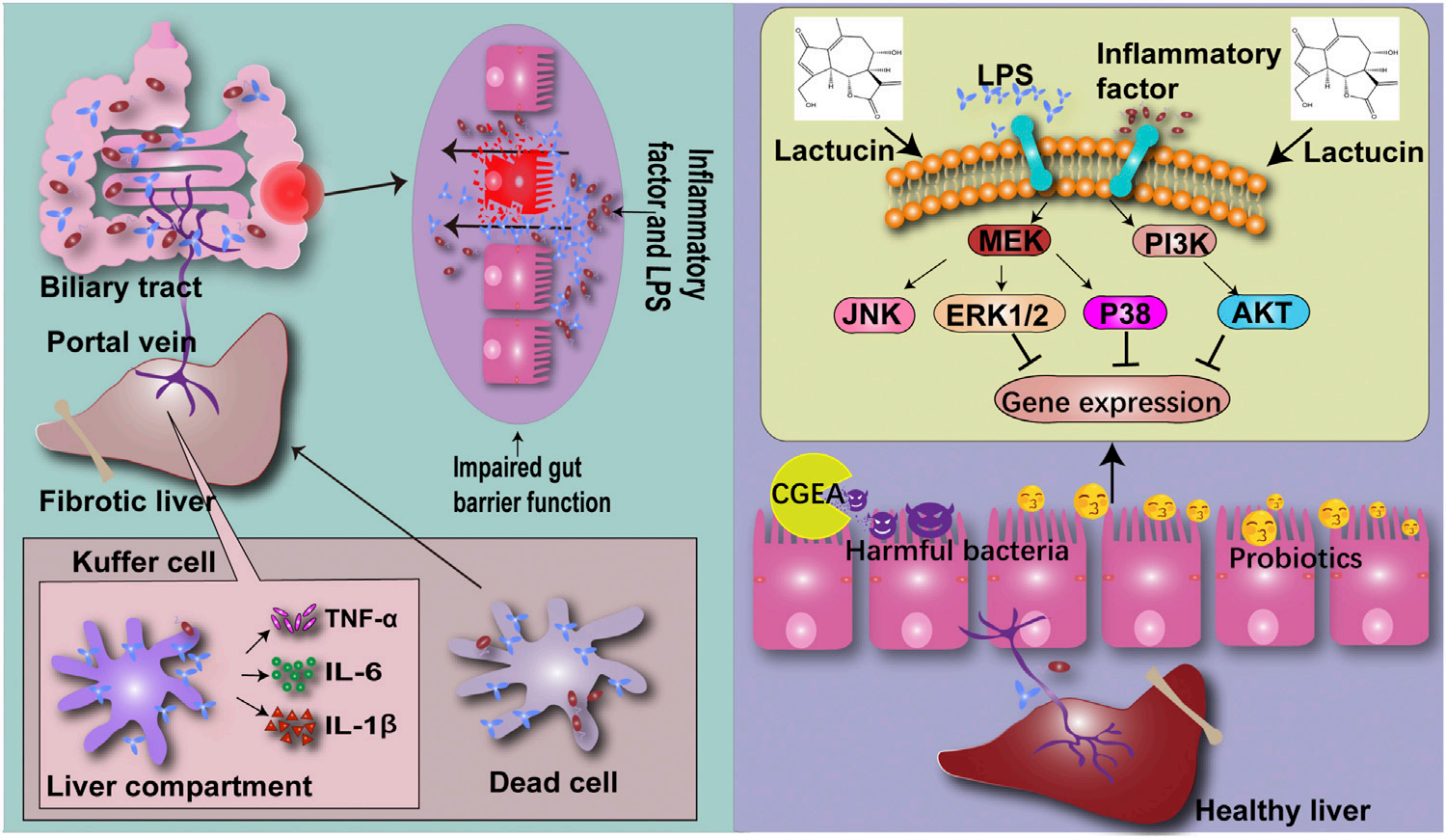
CG confers anti-inflammatory effects by inhibiting the phosphorylation pathway of MAPK signaling pathway, thereby improving the intestinal microenvironment and reducing the production of liver fibrosis through the “gut-liver axis”.

Taken together, our results indicated that CGEA can ameliorate liver fibrosis induced by 5% TNBS-50% ethanol solution. The mechanism of action may combine the modulation of ERK1/2, p38 and AKT signaling pathways with improvement of the “gut-liver axis” circulation to inhibit inflammation and thus exert an anti-fibrotic effect (Figure 10).

## Data Availability

The datasets presented in this study can be found in online repositories. The names of the repository/repositories and accession number(s) can be found in the article/Supplementary Material.

## GLOSSARY

AKT: (protein kinase B)
ALT: (Alanine aminotransferase)
Alb: (Albumin)
AST: (Aspartate aminotransferase)
CG: (Cichorium pumilum Jacq)
CGEA: (Ethyl Acetate Extract of Cichorium pumilum Jacq)
COX-2: (Cyclooxygenase-2)
DMSO: (dimethyl sulfoxide)
Elisa: (enzyme-linked immunosorbent assay)
ERK1/2: (Extracellular signal-regulated kinase)
IL: (Interleukin)
iNOS: (Inducible nitric oxide synthase)
JNK: (Jun-N-terminal kinase)
LDH: (Lactate dehydrogenase)
LPS: (Lipopolysaccharide)
MAPKs: (Mitogen activated protine kinase)
MTT: [3-(4,5-dimethylthiazol-2-yl)-2,5-diphenyltetrazolium bromide]
NO: (Nitic oxide)
p38: (p38 kinases)
qPCR: (Real-time Quantitative polymerase chain reaction)
γ-GT: (gamma-glutamyl transferase)
TNBS: (2,4,6-trinitrobenzene sulfonic acid)
TNF-α: (Tumor necrosis factor-α)
(BBL): Bifidobacterium
(MRS): DeMan-Rogosa-Sharpe
(RIPA): Radio Immunoprecipitation Assay
(BCA): Bicinchoninic Acid
