# Supplementary material for: Cichorium pumilum Jacq Extract Inhibits LPS-Induced Inflammation via MAPK Signaling Pathway and Protects Rats From Hepatic Fibrosis Caused by Abnormalities in the Gut-Liver Axis
Source: Front Pharmacol. 2021 Apr 29;12:683613. doi: 10.3389/fphar.2021.683613 (PMC8117150; doi:10.3389/fphar.2021.683613)
Supplement: Supplementary file 4 [file DataSheet3.DOCX]

**Supplementary Material**

Table 3. Chemical composition of CGEA

| NO | Name | Structure | Molecular  formula | Relative  molecular mass |
| --- | --- | --- | --- | --- |
| 1 | Lactucin |  | C_15_H_16_O_5_ | 276.28 |
| 2 | 11*β*,13-dihydrolactucin |  | C_15_H_18_O_5_ | 278.28 |
| 3 | 11*β*,13-dihydrosantamarin |  | C_17_H_26_O_2_ | 262.19 |
| 4 | 1*β*-hydroxy-eudesm-4-en-6*β*,7*α*,11*β*H-12,6-olide |  | C_15_H_22_O_3_ | 250.16 |
| 5 | Glandulosine D |  | C_18_H_25_O_6_ | 337.17 |
| 6 | Scorzoside |  | C_18_H_26_O_5_ | 322.18 |
| 7 | Glandulosine A |  | C_16_H_24_O_5_ | 296.16 |
| 8 | Glandulosine E |  | C_16_H_22_O_4_ | 278.15 |
| 9 | Sonchuside A |  | C_17_H_24_O_5_ | 308.16 |
| 10 | Scorzoside |  | C_21_H_30_O_8_ | 410.19 |
| 11 | 1-*O*-*β*-D-glucopyranosyl-5*α*,6*β*H-eudesm-3-en-12,6-olide |  | C_21_H_32_O_8_ | 412.21 |
| 12 | Glandulosine B |  | C_21_H_32_O_8_ | 412.21 |
| 13 | Glandulosine C |  | C_17_H_24_O_6_ | 324.16 |
| 14 | Lactucopicrin |  | C_23_H_22_O_7_ | 410.14 |
