# Supplementary material for: Cichorium pumilum Jacq Extract Inhibits LPS-Induced Inflammation via MAPK Signaling Pathway and Protects Rats From Hepatic Fibrosis Caused by Abnormalities in the Gut-Liver Axis
Source: Front Pharmacol. 2021 Apr 29;12:683613. doi: 10.3389/fphar.2021.683613 (PMC8117150; doi:10.3389/fphar.2021.683613)
Supplement: Supplementary file 9 [file DataSheet2.DOCX]

**
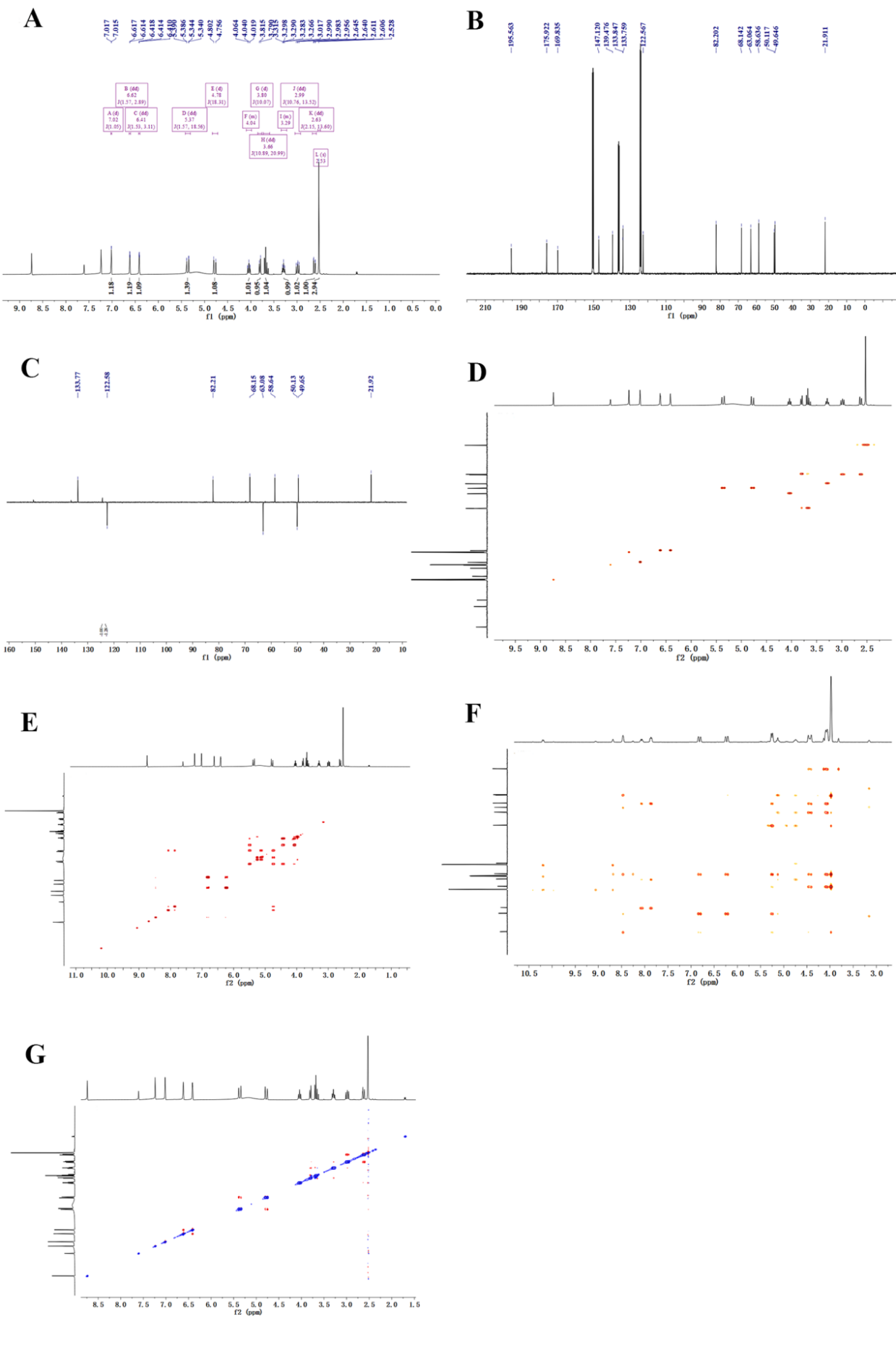
**

Sig 1. The chromatogram of Lactucin (4-hydroxy-9-(hydroxymethyl)-6-methyl-3- methylidene-4,5,9a,9b-tetrahydro-3aH-azuleno[4,5-b]furan-2,7-dione). (A) ^1^H NMR spectrum (CDCl_3_, 400 MHz). (B) ^13^C NMR spectrum (CDCl_3_, 100 MHz). (C) DEPT NMR spectrum (CDCl_3_, 100 MHz). (D) HSQC spectrum (CDCl_3,_ ^1^H: 400 MHz, ^13^C:100 MHz). (E) ^1^H-^1^H COSY spectrum (CDCl_3,_ ^1^H: 400 MHz). (F) HMBC spectrum (CDCl_3_, ^1^H: 400 MHz, ^13^C:100 MHz). (G) NOESY spectrum (CDCl_3,_ ^1^H: 400 MHz).
